# Tracing Translational Footprint by Ribo-Seq: Principle, Workflow, and Applications to Understand the Mechanism of Human Diseases

**DOI:** 10.3390/cells11192966

**Published:** 2022-09-23

**Authors:** Atefeh Bagheri, Artem Astafev, Tara Al-Hashimy, Peng Jiang

**Affiliations:** 1 Department of Biological, Geological and Environmental Sciences (BGES), Cleveland State University, Cleveland, OH 44115, USA; 2 Center for Gene Regulation in Health and Disease (GRHD), Cleveland State University, Cleveland, OH 44115, USA; 3 Center for Applied Data Analysis and Modeling (ADAM), Cleveland State University, Cleveland, OH 44115, USA; 4 Center for RNA Science and Therapeutics, School of Medicine, Case Western Reserve University, Cleveland, OH 44106, USA

**Keywords:** ribosome profiling, translation, human diseases, translation efficiency (TE)

## Abstract

RNA-seq has been widely used as a high-throughput method to characterize transcript dynamic changes in a broad context, such as development and diseases. However, whether RNA-seq-estimated transcriptional dynamics can be translated into protein level changes is largely unknown. Ribo-seq (Ribosome profiling) is an emerging technology that allows for the investigation of the translational footprint via profiling ribosome-bounded mRNA fragments. Ribo-seq coupled with RNA-seq will allow us to understand the transcriptional and translational control of the fundamental biological process and human diseases. This review focuses on discussing the principle, workflow, and applications of Ribo-seq to study human diseases.

## 1. Introduction

In the past few decades, deep-sequencing-based transcriptome profiling (e.g., RNA-Seq) has been the most utilized approach to investigate human diseases. Many disease transcriptomic markers and therapeutic targets have been found. However, evidence suggests that many human diseases are perturbated at the protein level but not at the transcription level. For example, a study on breast cancer found that the translational efficiency (the rate of mRNAs translating to proteins) in malignant cells tend to be more sensitive to the environment (e.g., under stress) than in nonmalignant cells [1], suggesting the perturbated translational machinery can be part of the complexity of pathogenesis. The questions, such as which mRNAs are prioritized by cancer cells to translate, cannot be addressed by the RNA-seq experiment alone. Ribo-seq (or ribosome profiling) is a deep-sequencing-based high-throughput method that measures the abundance of transcripts bounded by ribosomes. Given that most protein assays are low-throughput, Ribo-seq provides a unique technology that systematically measures translated transcripts. Current Ribo-seq protocols can achieve single-nucleotide resolution with high reproducibility [2,3]. Such resolution allows Ribo-seq to de novo identify mRNA open reading frame (ORF) [4] regions and detect potential cryptic translation events [5].

Although Ribo-seq is a valuable tool to investigate translational control in a broad context, only a few labs can apply this technology to their research. The bottleneck is due to both experimental and data analysis challenges. In this review, we discuss the principle, workflow, and data analysis tools of Ribo-seq. We also discuss how this unique technology can lead to novel findings for human diseases.

## 2. Principles of Ribosome Profiling and Technical Workflow

**Figure 1:**
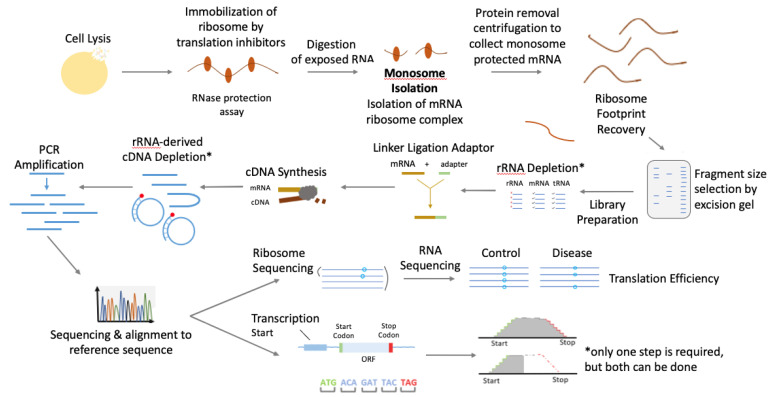
Schematic illustration of the current workflow of ribosomes. The experiment starts with cell lysis, which isolates and immobilizes the mRNA ribosome complexes, and is followed by nuclease digestion of mRNA sequences that are not protected by associated ribosomes. Purification of the mRNA fragments shielded by the ribosomes is then carried out, followed by standard deep sequencing protocols, such as library preparation.

The technical basis of Ribo-seq is that ribosome-bounded mRNA fragments are protected from nuclease digestion. Hence, purifying ribosome-bounded mRNA fragments coupled with high-throughput sequencing and a set of computational strategies (e.g., sequencing reads mapping to reference transcript sets) can be used to locate the ribosome footprint on mRNA transcripts. The detailed workflow can be found in Figure 1.

Specific key steps include:

(1) Freezing ribosomes on mRNAs to avoid ribosome run-off: After culturing the cells, they are treated with translation inhibitors [6]. Even though there are a wide variety of translation inhibitors (such as ribosome-targeting initiation, elongation, and termination inhibitors; inhibitors of initiation and elongation factors; and the inhibitors of cell signaling pathways involved in translational control), arguably the most commonly used for ribosomal footprinting are the classical elongation inhibitors, such as cycloheximide, anisomycin, emetine [7]. These inhibitors, however, have to be used with caution, considering their potential artifact effects in ribosomal distribution, which are specific to cell/organism type, cell growth conditions, and the contents of lysis buffer [8]. For instance, treatment with cycloheximide results in a larger ribosomal distribution at the 5′ coding end of the transcript. The effect is more pronounced in S. Cerevisiae and in shorter transcripts [6,7,8]. Alternatively, if the goal is to specifically inhibit ribosomes at their initiation sites, inhibitors specific to de novo initiation ribosomes are used: harringtonine and lactimidomycin are examples of inhibitors with such activity [6,7]. In light of potential challenges associated with the use of translation inhibitors, in situ detergent lysis of cells is advised where applicable [6]. Flash-freezing and cryogenic pulverization may be suitable for specific cell and tissue types (mainly animal cells and tissues) [2,6,8].
(2) Ribonuclease digestion breaks down unprotected regions of mRNA: Ribonucleases are responsible for the breaking down and degradation of exposed mRNA, except for ribosome-protected fragments (RPFs). The precision of this step is dependent on buffer conditions, where a lower concentration of sodium (150–200 mM) and magnesium (5mM-10mM) is preferred for optimally uniform digestion by the nuclease [6].
(3) mRNA–ribosome complex recovery and RNA purification: After ribonuclease digestion, the mRNA–ribosome footprint complexes are recovered using sucrose density fractionation or sedimentation through a sucrose cushion [6]. Alternatively, mRNA–ribosome complexes can be retrieved by gel filtration [9] or affinity purification with the use of an epitope tag added to the ribosomes, which is essential in profiling specific cell types [10,11]. The resultant ribosomal pellet is resuspended in Trizol/Qiazol reagent, and the protected RNA fragments are then purified using a spin column, such as miRNeasy kit [6]. Subsequently, the isolated RNA is separated by polyacrylamide gel electrophoresis to isolate the RNA fragments of 26–34 nt length corresponding to the RPFs.
(4) Library generation and deep sequencing: Following RPF purification, it is essential to deplete the sample of rRNA. Ribosomal RNA is the most predominant RNA, making up about 80% of cellular RNA in the cell, and its removal is essential because the abundance of rRNA leads to fewer RPFs being sequenced and fewer mapping reads. This, as a result, reduces the useful size of the library. rRNA can be depleted using kits, such as Ribo-zero Plus or Legacy Ribo-zero (Illumina, San-Diego, C), which use anti-rRNA DNA probes with subsequent removal of rRNA by enzymatic digestion (RNase H) or by streptavidin affinity pull-down assay. When RPFs are depleted of rRNA, the fragments need to be ligated or fused to adapters [6]. RPFs can be tailed with a poly-A polymerase, or else a single-stranded RNA linker of a known sequence can be ligated to the 3′-end of the RPF [12]. The RNA fragments are then converted into complementary DNA by the enzyme reverse transcriptase. The cDNA later serves as a template for exponential amplification using PCR. This library can then be sequenced to generate a bioinformatics library containing RPF sequenced reads [2,13]. As an alternative strategy, rRNA depletion can be carried out after the reverse-transcription-step cDNA is circularized. The rRNA subtraction oligos (biotinylated sense-strand oligonucleotides against rRNA-derived cDNA) are added. rRNA-derived cDNA fragments are removed using a streptavidin affinity assay. The remaining cDNA fragments represent the non-rRNA RPFs, which are further PCR-amplified and sequenced [6]. Mapping these sequenced RPFs to the transcriptome provides a snapshot of translation that reveals transcriptome wide the positions and densities of ribosomes on individual mRNAs.
(5) Data analysis: The upstream steps of Ribo-seq analysis are similar to the standard RNA-seq data analysis workflow [14], including read mapping to transcripts. The downstream Ribo-seq data analysis includes calculating transcript translational efficiency when the data are paired with RNA-seq, identifying differentially translated transcripts, de novo identifying transcript open reading frame (ORF), and detecting potential translational pausing events [6].

## 3. Ribo-Seq Provides a More Accurate and Closer View of Protein Expression than RNA-Seq

RNA-seq measures the total amount of mRNA. It is a method used to measure transcriptional regulation. Ribo-Seq, on the other hand, can be used to measure translated mRNAs, providing a more accurate and closer view of protein expression than RNA-Seq [15]. Therefore, ribosome footprint abundances correlated better with genome-wide protein levels than RNA-seq data.

## 4. Using Ribo-Seq to Estimate Protein Abundance

Technically, Ribo-Seq is used to estimate the abundance of translated mRNAs but not the protein expression levels. A study using a paired shotgun proteomics experiment (liquid chromatography with tandem mass spectrometry (LC-MS-MS) with the Ribo-seq experiment found that the translated mRNAs estimated by Ribo-Seq were correlated fairly well with LC-MS-MS-estimated protein abundance, with a Pearson correlation coefficient ranging from 0.483 to 0.664 [16]. This is consistent with the commonly accepted notation that most ribosome-bound mRNAs can translate to proteins. However, we should be aware that this correlation only holds true under certain conditions and may not be true for other conditions. This is because mass spectrometry cannot separate newly synthesized versus pre-existing proteins, while Ribo-seq measures “to be translated mRNAs”. Technically, Ribo-seq may be more correlated with newly synthesized proteins than total proteins (newly synthesized plus pre-existing proteins).

## 5. Computational Methods and Tools to Analyze Ribo-Seq Data

The computational methods for Ribo-seq data analysis can be classified as upstream methods (raw data processing: quality control and reads mapping) and downstream methods (including but not limited to quantification of ribosome-bounded transcripts, translated ORF identification, and differential translation analysis). In addition, there are two computational challenges:

(a) Identifying ribosomal A and P sites on ribosome-protected mRNA fragments: Ribosomes have three sites (A, P, and E) where tRNAs can bind to an mRNA. The A site accepts an incoming tRNA, and the P site holds a tRNA that carries a growing polypeptide. The E site is where a tRNA goes after it is empty. The A, P, and E sites are three nucleotides apart. For a ribosome-protected fragment, the information about where the A, P, and E sites are located is removed during the digestion step. Hence, identifying these sites within the ribosome footprint is fundamentally important for understanding translation at the codon scale. Multiple computational methods have been proposed to address such problems. The fundamental basis of these methods is based on the fact that only the P site is permitted to occupy the start codon during translation initiation, and only the A site is permitted to occupy the stop codon during termination. Therefore, the relative position of A and P sites in ribosome-protected fragments can be determined by Ribo-seq reads mapped at the start and stop codons [2,17]. One problem with these approaches is that RNase digestion is highly stochastic, and, thus, the ribosome-protected fragment length varies within the same Ribo-seq experiment. Hence, it is technically hard to determine A or P sites for a fragment library with a spectrum of fragment length. To solve this problem, a study utilized the fundamental biological fact that the A site on ribosome-protected fragments must reside within the CDS region to create a function that maximizes the number of fragments with the A site falling into the CDS region.
(b) Data normalization to local ribosome profiling read density: The Ribo-seq read distribution on a mapped transcript reference is typically characterized as alignment gaps, sporadic high-density peaks due to technical artifacts, and ribosome pauses [18]. These fluctuations substantially generate challenges for downstream data analysis, limiting the ability to characterize factors influencing global ribosome read density accurately. Hence, data normalization for local ribosome read density is necessary before performing downstream analysis. The most intuitive approach is normalizing the local Ribo-seq signal by the average signal across the coding region [19]. These approaches are based on the assumption that the ribosome footprint is smoothly distributed to different codons. However, such approaches are very sensitive to high-density peaks (rare in RNA-seq but high frequency in Ribo-seq due to ribosome pausing) and performed poorly for transcripts of low ribosome read coverage. A simple but robust computational method (“RUST”) was proposed to reduce this local Ribo-seq read density bias to solve these problems. The RUST method converts ribosome footprint densities into a binary unit function (Heaviside step function). Each codon is given a score of 1 or 0 depending on whether the footprint density at this codon exceeds the average for the corresponding ORF.

A comprehensive overview of computational methods and tools can be found in Table 1:

## 6. Footprints and Translation Efficiency

Footprints or mRNA-protected fragments originate from the translated region of mRNAs, which result from the nuclease digestion of ribosome complexes converted to a library for deep sequencing. They are approximately 28–30 nucleotides long. Each footprint has a characteristic sequence that can be mapped on the transcriptome, allowing us to identify novel coding regions on transcripts previously thought to be non-coding at the moment of cell lysis. This also allows for the analysis of N-terminal protein heterogeneity and termination codon readthrough, which results in C- terminal protein extension. Since millions of RPFs are sequenced in parallel, we can obtain very detailed, quantitative information about the pool of cellular ribosomes and how they translate mRNA. This technique can be applied to many organisms, from bacteria to metazoans [2,45]. Essentially, RPFs are a proxy for protein synthesis rates: typically, the more RPFs map to a given transcript, the more proteins are synthesized from this transcript in a given timeframe. The distribution of RPFs in the coding region can also be used to identify codon-specific translation defects or regions in the transcriptome that are difficult to translate. Extensive RPF accumulation of the RPFs specific to a particular codon may indicate ribosome stalling.

The ribosome profiling technique involves measuring translational efficiency (TE) by comparing the levels of ribosome-associated mRNA footprints to the total mRNA for each gene. The obtained snapshot of ribosome occupancy on the coding region has often been used to estimate relative changes in translation efficiency under different growth conditions. We can calculate translational efficiency (TE) to learn which transcript translates better than others. High TE scores are mostly correlated with efficient translation. Changes in TEs between different conditions typically indicate translational regulation [46].

These are just some of the many different applications of ribosome profiling. Novel adaptations of the protocol are regularly introduced, allowing us to analyze the more detailed aspects of translation.

## 7. Ribo-Seq Provides a Unique High-Throughput Method to Understand Disease Mechanisms at the Translational Level

It is well-known that a significant barrier from mRNA to protein is translational efficiency. In cancer cells, specific mRNA transcripts are more likely picked up by translational machinery than others compared to the cohort [1]. Identifying such disease signatures is technically challenging due to the relatively low throughput of protein assays. Ribo-seq paired with RNA-seq provides a high-throughput approach to systematically investigate the disease perturbated translational footprint on mRNAs. Table 2 listed human diseases associated or partially associated with translational machinery.

Ribo-seq allows for the investigation of the mechanism of human diseases at the level of translational control. This layer of information cannot be easily obtained from RNA-seq because RNA-seq is used to profile the transcription process but not the translation process. Hence, a gene that shows differences at the translational level (e.g., protein level changes) but not at the transcriptional level in human diseases will not be detected by RNA-seq. Ribo-seq provides a tool that allows us to explore translation control in a high-throughput manner.

## 8. Ribosome Collisions

The standard Ribo-seq protocol is used to study ribosome footprints during translational elongation via targeting single ribosome-bounded fragments by fragment size selection. However, several studies observed larger ribosome-protected mRNA fragments, suggesting that more than one ribosome (disome and trisome) may co-exist during translational elongation [53,54]. Several studies used Ribo-seq protocols to explore these ribosome collision events in order to investigate their biological significance, suggesting that ribosome collisions are associated with translational stalling [54] and co-translational protein folding [53].

## 9. Limitations and Potential Solutions

(a) Estimation of global changes in translation: The standard Ribo-seq protocol only estimates the relative abundance of ribosome-bounded mRNAs but cannot quantify absolute global changes in translation. This is because, for a particular translated transcript, the sequencing reads from Ribo-seq depend on sequencing depth, open reading frame (ORF) length, and competition with other translated transcripts. After data normalization to control factors, such as read depth and ORF length, the number of mapped reads of a particular transcript will be primarily determined by the relative abundance. Hence, the information about the absolute abundance of transcripts is lost. A possible solution is to add spike-in before the sequencing sample preparation step. Spike-in mRNAs are synthetic nucleic-acid sequences added to the sequencing library. Since we know the spike-in concentration, the absolute abundance of each translated mRNA can be inferred by comparing transcript read counts with spike-in read counts. A study using this strategy can successfully quantify the absolute global change in translation [55].
(b) Measuring translation in individual cells: The standard Ribo-seq protocol is designed for measuring translation at the bulk mRNA level (an ensemble of different cell types). Emerging single-cell RNA-seq (scRNA-seq) technology allows the investigation of the heterogeneity of individual cells. However, due to the amplification of a low amount of RNAs, scRNA-seq suffers a high dropout effect (certain RNAs are missed during amplification) due to technical problems. Moreover, a mixture of technical artifacts with actual biological variant cells makes this even more complicated [56]. Ribo-seq at the single-cell level is more technically challenging than RNA-seq. This is because (a) not all mRNAs are bounded by ribosomes, and (b) the extra pulling out ribosome-bounded RNAs may lose a certain number of mRNAs. Hence, the expected start material of single-cell Ribo-seq is lower than scRNA-seq. To solve this problem, a modified Ribo-seq protocol is needed. A recent study modified the existing Ribo-seq protocol to increase the sensitivity of the protocol, allowing for the profiling of Ribo-seq at the single-cell level [57].

## Figures and Tables

**Table 1:** A list of computational tools to analyze Ribo-seq data.

	Methods and Website	Features
Quantification of Ribosome-bounded transcripts	riboSeqR: An R/Bioconductor package that provides a set of programs for processing and visualization of Ribo-seq data. https://ribogalaxy.genomicsdatascience.ie/ [20]	Provides visualization of data at sub-codon resolution in the context of single transcripts.
	Plastid: A user-friendly, generalized analytical pipeline tool that enables users to manipulate data nucleotide by nucleotide robustly and easily and that is not limited to specific experimental regimes or analytical workflows. https://plastid.readthedocs.io [21].	Extensibility and flexibility across assays while remaining user friendly.
	RUST: A smoothing transformation-based approach for Ribo-seq normalization in the presence of heterogeneous noise. https://lapti.ucc.ie/rust/ [22].	Performs better in presence of sporadic heterogeneous noise than the previous methods.
	mQC: A tool for visualizing quality and data exploration after mapping. https://github.com/Biobix/mQC [23].	Applies the P site offsets before plotting to inspect ribosomal framing and triplet periodicity more elaborately than other existing tools.
	GWIPS-viz: An online genome browser for checking quality measures or discovering authentic new information from ribosome profiling data. https://gwips.ucc.ie/ [24].	A Ribo-seq genome browser for data visualization
	RiboVIEW: A computational pipeline for visualization, quality control, and statistical analysis of ribosome profiling data. https://github.com/carinelegrand/RiboVIEW [25].	Focuses on checking quality measures.
	Trips-Viz: A graphical tools for exploring properties of collection of ORFs. https://trips.ucc.ie/ [26].	Provides visualization of data at sub codon resolution in the context of single transcripts.
Translated ORF identification	RibORF: A support-vector-machine-based classifier to determine which RNAs are translated based on read distribution features. https://github.com/zhejilab/RibORF [27].	Helps to discern between the RNAs that are genuinely translated and those that are not associated with ribosomes.
	RiboTaper: A multitaper spectral-based approach for comprehensive de novo identification of actively used ORFs from Ribo-seq data. https://ohlerlab.mdc-berlin.de/software/RiboTaper_126/ [28].	General applicability and excellent performance in reconstructing full set of ORFs (coding and non-coding).
	ORF-RATER: An experimental and analytical framework based on linear regression for identification and quantification of translation. https://github.com/alexfields/ORF-RATER [29].	Helps in comprehensive interpretation of ribosome profiling data due to flexibility of the linear regression model.
	SPECtre: A memory-efficient analytical tool (spectral coherence-based classifier) with more accuracy for detecting active translation. https://github.com/mills-lab/spectre [30].	Optimization runtime and memory for accurate investigation of translation.
	riboHMM: A mixed hidden Markov model-based approach to accurately infer translated sequence. https://github.com/rajanil/riboHMM [31].	Infers novel translated sequences with a focus on short CDSs (<100 amino acids).
	RpBp: An unsupervised Bayesian approach for predicting translated ORFs. https://github.com/dieterich-lab/rp-bp [32].	Improves predictions while maintaining distributions through the entire process.
	PRICE: A software pipeline including all steps necessary to identify and score codons and ORFs starting. https://github.com/erhard-lab/gedi/wiki/Price [5].	Modeling the experimental noise to accurately resolve overlapping sORFs.
	RiboWave: A computational method using wavelet transform to remove noise for detecting actively translated (ORFs) and dynamic cellular translation. https://github.com/lulab/Ribowave [33].	Indicates low-quality reads to improve the performance of ORF prediction.
	RiboCode: A method for evaluating the active translation mainly based on the 3 nt periodicity. https://pypi.org/project/RiboCode/ https://github.com/xryanglab/RiboCode [34].	Higher efficiency and accuracy for de novo annotation and characterization of the translatome with ribosome profiling data.
Differential translation analysis Identification of A and P site location	Riborex: A linear model-based tool for identification of differential translation from Ribo-seq data. https://github.com/smithlabcode/riborex [35].	Faster than all existing methods and employs robust software implementations for the underlying statistical calculations.
	Anota: An R/Bioconductor package that implements analysis of partial variance (APV) to identify differential translation. https://github.com/ChrOertlin/anota2seq/ [36].	Using APV instead of log ratio approach for detecting translation changes.
	Babel: An errors-in-variables regression model-based framework to compare ribosome associations within and between conditions based on an errors-in-variables regression model. https://github.com/olshena/babel [37].	Model is more flexible and combines P-values across independent tests.
	RiboDiff: A linear model-based framework for detecting changes of mRNA translation efficiency across experimental conditions. http://bioweb.me/ribodiff http://github.com/ratschlab/ribodiff [38].	Facilitating comparisons of RF abundance by taking mRNA abundance variability as a confounding factor.
	Xtail: An analysis pipeline to detect differentially translated genes. https://github.com/xryanglab/xtail [39].	A more sophisticated method for domination on limitations, such as high-false discoveries and low sensitivities.
	RiboProfiling: An R/Bioconductor package that provides a full pipeline to cover all key steps for facilitating the analysis of Ribo-seq experiments and ribosome footprints. https://github.com/alenzhao/RiboProfiling [40].	Utilizes multiple R packages to handle datasets easily.
	RiboA: A user-friendly web application that identifies A site locations and generates read density profiles. Website: https://a-site.vmhost.psu.edu/ https://github.com/obrien-lab/aip_web_docker [41].	The most accurate identifier compared to other tools.
	riboWaltz: An R package for the identification of the ribosome P site, analysis, and visual inspection of ribosome profiling data. https://github.com/LabTranslationalArchitectomics/RiboWaltz [42].	Addresses issue of time limitation and data preprocessing.
	RiboToolkit: A freely available, web-based service to centralize Ribo-seq data analyses, codon occupancy, and translation efficiency analysis. http://rnainformatics.org.cn/RiboToolkit/ [43].	Addresses the lacking integrated tool and easy-to-use integrated tool to analyze Ribo-seq data.
	RiboTools: An open-source Galaxy tool used to evaluate codon occupancy at a specific ribosome site and for translation readthrough events. https://testtoolshed.g2.bx.psu.edu/view/rlegendre/ribo_tools [44].	Facilitates complete qualitative analysis.

**Table 2:** Ribo-seq identifies human disease mechanisms at the translational level.

Diseases	Major Findings via Ribo-Seq
Breast Cancer	The translational efficiencies tend to have higher variations in malignant cells than controls under perturbations, such as condition changes or stress. [1]
Prostate Cancer	Uncovering major translations by mTOR kinase and revealing the collection of genes involved in a different step of the cell cycle allows improvement in understanding of how cancerous translation operates cancer-specific cell behavior [47].
Brain Tumor	Genes specific to transformed cells are highly translated, but their translation efficiencies are low compared with the normal brain. Furthermore, the upregulated pathways found in tumor-associated cells are most closely associated with the mesenchymal subtype [48].
Human Leukocyte Antigen (HLA)	Significantly higher positive correlation between HLAIp sampling searched against Ribo-Seq and the translation rate than the overall RNA abundance. Identification of additional upstream ORFs or other unannotated ORFs that are not included in canonical annotation but still show periodic footprint of translation [49].
Diamond–Blackfan Anemia (DBA)	Molecular lesions underlying DBA reduce ribosome levels in hematopoietic cells and this reduction causes impaired translation of a subset of mRNAs. Furthermore, translational perturbations in DBA impair lineage commitment in HSPCs [50].
Fragile X Syndrome (FXS)	Reveals diverse changes in gene expression in Fmr1 KO hippocampus [51].
Amyotrophic Lateral Sclerosis (ALS) and Frontotemporal Dementia (FTD)	Identification of a novel function of TDP-43 (has a central role in neurodegenerative diseases) as an mRNA-specific translational enhancer, which enhances translation of Camta1 and Mig12 mRNAs via their 5′UTRs and specific 3′UTR region for Dennd4a [52].

## Data Availability

Not applicable.
